# Beyond the host: Unveiling the independent microbiome of equine gastrointestinal nematodes

**DOI:** 10.1371/journal.pone.0339596

**Published:** 2026-02-10

**Authors:** Fabio Gentilini, Tolulope Grace Ogundipe, Maria Elena Turba, Noemi Romagnoli, Carlotta Lambertini, Claudia Pollera, Paola Cremonesi, Laura Stancampiano

**Affiliations:** 1 Department of Veterinary Medical Sciences (DIMEVET), University of Bologna, Ozzano dell´Emilia (Bo) Italy; 2 Genefast srl, Forlì, Forlì-Cesena, Italy; 3 Department of Veterinary Medicine and Animal Sciences (DIVAS), University of Milan, Lodi, Italy; 4 Institute of Agricultural Biology and Biotechnology (IBBA), National Research Council (CNR), Lodi, Italy; Camerino University: Universita degli Studi di Camerino, ITALY

## Abstract

Gastrointestinal nematode infections significantly impact equine health and welfare, with rising anthelmintic resistance demanding alternative control strategies. Emerging evidence suggests that parasitic nematodes harbour distinct microbiomes, potentially influencing host-parasite dynamics and parasite survival. This study aimed to characterize and compare the microbiomes of equine gastrointestinal nematodes and their hosts, focusing on differences in composition, diversity, and core microbiota structure across different intestinal sites, nematode subfamilies, and sexes. Faecal and nematode samples were collected from equids (*Equus caballus* and *Equus asinus*) at slaughterhouses. DNA was extracted, and the V3-V4 regions of the 16S rRNA gene were amplified and sequenced using the Illumina iSeq 100 platform. Bioinformatic analyses were performed with QIIME2 and MicrobiomeAnalyst, and statistical comparisons employed PERMANOVA, LEfSe, and alpha and beta diversity metrics. Nematodes exhibited a distinct microbiome dominated by Firmicutes, Proteobacteria, Bacteroidota, Verrucomicrobiota, and Actinobacteriota, differing significantly from the faecal microbiota. Alpha diversity analyses revealed lower richness in nematodes, while beta diversity indicated distinct community structures (p = 0.007). Microbial composition varied by gastrointestinal site, nematode subfamily, and sex. Proteobacteria were consistently enriched in nematodes, particularly in the caecum. Core microbiome analysis identified exclusive nematode-associated taxa such as *Fusobacterium*, *Mesorhizobium*, and *Mycoplasma*. Equine gastrointestinal nematodes harbour independent and structured microbiomes, distinct from those of their hosts. These findings underscore the ecological specialization of nematodes and highlight the potential of targeting parasite-associated microbiota for novel control strategies.

## Introduction

Parasitic infections, particularly gastrointestinal nematode infestations, pose a persistent challenge in equine veterinary medicine, significantly affecting animal welfare, growth, and productivity [1,2]. These infections compromise the immune system and cause nutritional deficiencies, predisposing animals more susceptible to secondary infections [2,3]. Such health compromise leads to severe health consequences and substantial economic losses. Traditional anti-parasitic treatments, though initially effective, have become increasingly unreliable because of the rising prevalence of anthelmintic resistance, particularly to commonly used drugs like ivermectin and albendazole [4,5]. This resistance underscores a pressing need for novel and sustainable approaches to parasite management.

Recent advancements in genomic sequencing and microbiome profiling have begun to elucidate key aspects of host-parasite interactions, suggesting that microbial-mediated mechanisms of immune modulation, nutrient competition, and parasite fitness may play critical roles within host environments [6,7]. Emerging evidence indicates that parasitic infections strongly influence the composition of the gut microbiome, a diverse ecosystem of bacteria, fungi, and other microorganisms essential for immune function, nutrient absorption, and pathogen defence [8,9]. Nematode infestations/colonization of the gut have been associated with significant influence on microbial composition, often resulting in dysbiosis, a state of microbial imbalance that can exacerbate parasitic infections and compromise overall host health [10,11]. By enabling detailed profiling of these microbial communities, microbiome analysis offers valuable insights into the relationships between the host and its parasitic burden.

This study first aims to compare the microbiome of equine faecal samples with that of intestinal nematodes collected from the same portion of the gastrointestinal tract from which the faecal microbiome originates, in order to determine whether nematodes maintain a microbiome that is distinct from that of the host gut [12,13]. Building on this, we further investigate whether the composition of the strongyle microbiome varies according to the gastrointestinal compartment (caecum vs ventral colon), the sex of the nematode, and the subfamily (large strongyles [Strongylinae] vs small strongyles [Cyathostominae]). In this way, the study seeks to characterise both the general differences between host and parasite microbiomes and the more specific patterns within nematode populations [12,13].

## Materials and methods

### Sample collection and DNA extraction

Faecal and nematode samples (the nematode samples were collected per host to capture intra-host parasite diversity; these are treated as nested replicates rather than independent samples) were collected from *Equus caballus* (n = 6) and *Equus asinus* (n = 1) at a slaughterhouse in the Emilia-Romagna region of Italy, totaling 50 samples (43 nematodes and 7 faecal samples) as shown in S1 Table. Approximately 100 grams of faeces were collected from the ventral colon and caecum using sterile spatulas to avoid cross-contamination. Samples were immediately transferred into sterile containers and transported in cooling boxes to the laboratory to maintain microbial integrity within a few hours. It is noteworthy that strongyles and cyathostomins were the predominant nematodes found in the selected host from the slaughterhouse, we restricted our microbiome analysis to these species. Ascarids were present at very low numbers and were therefore excluded from analysis

Nematodes were isolated by decanting with physiological water and then washed in distilled water and sexed under a stereomicroscope. The anterior region was excised with a sterile scalpel and preserved in 70% ethanol for identification; the posterior region was stored at −20 °C for future metagenomic analyses. Cephalic portions were mounted on slides, cleared in lactophenol, and morphologically identified following Lichtenfels et al. [14]. DNA extraction was performed using the Norgen Stool DNA Isolation Kit. For faecal samples, up to 200 mg of material was combined with Lysis Buffer L and vortexed, followed by the addition of Lysis Additive A. After thorough mixing, bead tubes were vortexed horizontally at maximum speed and centrifuged at 14,000 RPM for 2 minutes. The clean supernatant was collected, combined with Binding Buffer I, incubated on ice, and centrifuged again to remove debris. For nematodes, the extraction protocol included an additional overnight incubation at 56°C after the addition of Lysis Additive A to ensure complete tissue lysis. Subsequent steps mirrored those used for faecal samples. After lysis, lysates were mixed with 70% ethanol, vortexed, and applied to spin columns for DNA purification. The extracted DNA was eluted following the manufacturer’s guidelines and stored at −20°C for subsequent analyses.

### PCR amplification of the 16S rRNA gene and library preparation/pooling

The amplification of the V3-V4 region of the 16S rRNA gene was carried out following the extraction of DNA from the samples. Each PCR reaction was set up in a total volume of 25 µL, comprising 22.5 µL of a master mix and 2.5 µL of the diluted DNA template (1:10 dilution). The master mix was prepared with 12.5 µL of 2X Phusion PCR Buffer, 1.25 µL of the 16S amplicon PCR forward primer Pro341F and an overhang adapter sequence (5′- TCGTCGGCAGCGTCAGATGTGTATAAGAGACAG – CCTACGGGNGGCWGCAG −3′), 1.25 µL of the 16S amplicon PCR reverse primer Pro805R and an overhang adapter sequence (5′- GTCTCGTGGGCTCGGAGATGTGTATAAGAGACAG – GACTACHVGGGTATCTAATCC −3′), and 7.5 µL of nuclease-free water to ensure optimal reaction conditions. The PCR amplification was executed with Phusion High-Fidelity DNA polymerase under a controlled thermal cycling protocol, including an initial denaturation at 98°C for 30 seconds, followed by 25 cycles consisting of denaturation at 98°C for 5 seconds, annealing at 60°C for 10 seconds, extension at 72°C for 30 seconds, and a final extension at 72°C for 30 seconds. Each PCR run includes positive and negative controls to monitor contamination, ensure result reliability and confirm the success of the amplification process.

Following the PCR amplification, Illumina sequencing adapters and dual-index barcodes were added into the amplicons using the Nextera™ XT DNA Index Kit, enabling the identification of individual samples within the multiplexed sequencing run.

The resultant libraries were then normalized and pooled in preparation for sequencing on the iSeq 100 System with spiking-in 5% PhiX Control v3 (Illumina). The libraries were loaded into a prefilled reagent cartridge for sequencing run.

### Bioinformatics analysis

FASTQ files were analysed using a QIIME2 bioinformatic workflow run within a Conda environment (https://conda.org/). Demultiplexed paired-end sequences were imported as a QIIME2 artifact containing sequence data and metadata (sex, nematode type, biome, sampling location, and date). The primers, indexes and adapters were trimmed.

High-quality sequences were then processed through the denoising approach of DADA2 algorithm via the QIIME2 dada2 denoise-single. This critical step resulted in the generation of a feature table, representative sequences, and detailed denoising statistics, with truncation lengths established based upon the visual information of the distribution of sequence qualities at each position and taking into account a threshold of Q30 median value in the sequence data. The truncation was performed at about 135 bp for the forward read and 115 bp for the reverse read to maintain stringent quality control. The number of the retained sequences were checked.

Amplicon Sequence Variants (ASVs) generated from the denoised sequences were taxonomy assigned using the sklearn classifier and the SILVA 138 database as the taxonomic reference. Two samples (both female cyatostominae, on from caecum and the other from ventral colon) yielding less than 10K reads were excluded by subsequent analysis after filtering.

The resulting ASVs were subsequently imported into MicrobiomeAnalyst (microbiomeanalyst.ca) for advanced visualization and diversity analysis.

The full workflow was set up by analysing replicates of the ZymoBiomics gut Microbiome Standard and the Standard GUT and the ZymoBiomics Microbial Community Standard (Zymo Research).

### Statistical analysis

Alpha diversity metrics, such as Chao1, Shannon, and Simpson, were evaluated using the “features” taxonomy level and the paired Mann-Whitney tests for group comparisons. The beta diversity of microbial communities was calculated using Bray-Curtis dissimilarity, while differences between groups were assessed through Permutational Analysis of Variance (PERMANOVA). Principal Coordinate Analysis (PCoA) was employed for visualizing community structure, and the Linear Discriminant Analysis Effect Size (LEfSe) was utilized to assess differences in the relative abundances of individual microbial species between groups. The heat tree analysis leverages the hierarchical structure of taxonomic classifications to quantitatively (using the median abundance) and statistically (using the non-parametric Wilcoxon Rank Sum test) depict taxonomic differences between microbial communities. Finally, correlation networks were constructed using SECOM 1 to estimate pairwise microbial associations from compositional data.

### Ethics statement

All procedures adhered to institutional and national guidelines for the ethical use of animal-derived materials.

## Results

The analysis of taxonomic profiles and microbiota composition was conducted using the V3-V4 region of the 16S rRNA gene, sequenced across 43 nematode and 7 faecal samples. The sequencing effort generated 5,694,825 reads in total, averaging 113,897 reads per sample, with read counts ranging from 3,154–851,084. Following data quality filtering, denoising and chimera exclusion, reads were further processed to remove low quality or uninformative features as singletons and low variant features, yielding 4,564,904 features (mean, max, min = 91,299, 2,635, 713,441, respectively) accounting 2,177 operational taxonomic units (OTUs) retained from the initial 4,385, annotated using the SILVA 138 database. (Table 1; S1 Fig) Finally, to capture nearly all samples (n = 49; 7 faeces and 42 nematodes), the features were rarefied to 7,500 (S1 Fig).

**Table 1:** Summary of sequencing data processing steps for nematode and faecal samples.

Biome	n	input reads	Filtered	Percentage of input passed filter	Denoised	Non-chimeric	Percentage of input non-chimeric	Features after filtering for singletons and low variance features
Nematodes	43	114,159	108,839	96.6%	108,448	103,193	93.3%	92,014
Faeces	7	112,283	108,215	96.3%	107,204	97,218	87.3%	86,901

The table reports the average number of sequencing reads obtained at each step of the data processing pipeline. Input reads: total raw sequencing reads obtained from the sequencer; Filtered reads: reads retained after quality filtering; Denoised reads: reads processed through a denoising algorithm; Non-chimeric reads: reads remaining after removing chimeras—artefactual sequences formed by the joining of two or more parent sequences using chimera detection algorithms. Normalized reads.

### Microbial composition of faecal and nematode samples

**Fig 1:**
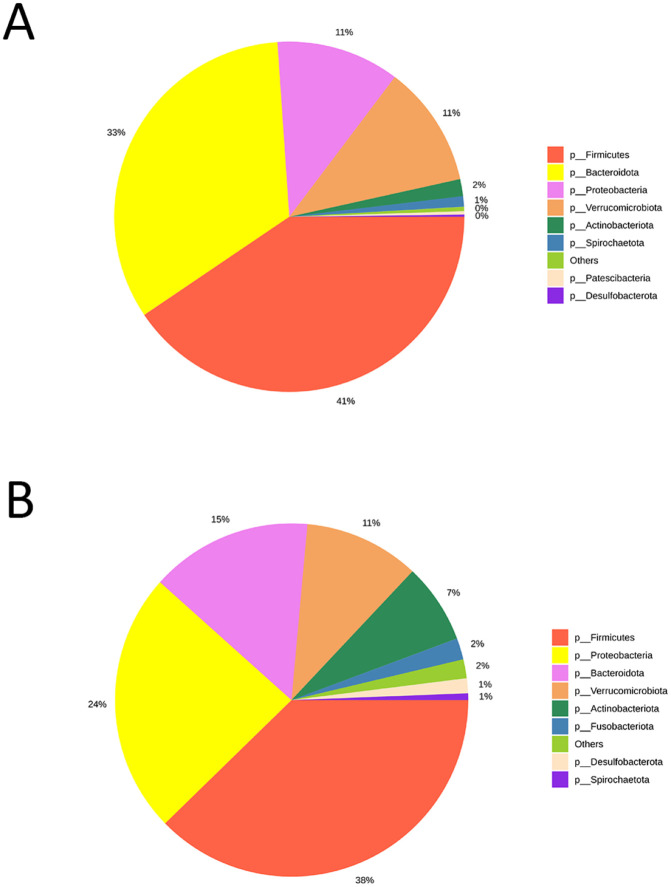
Pie chart illustrating the relative abundance of microbial composition at the phylum level identified in both faecal (A) and nematode (B) samples, facilitating a comparative analysis of microbial community structures across the two biomes.

**Fig 2:**
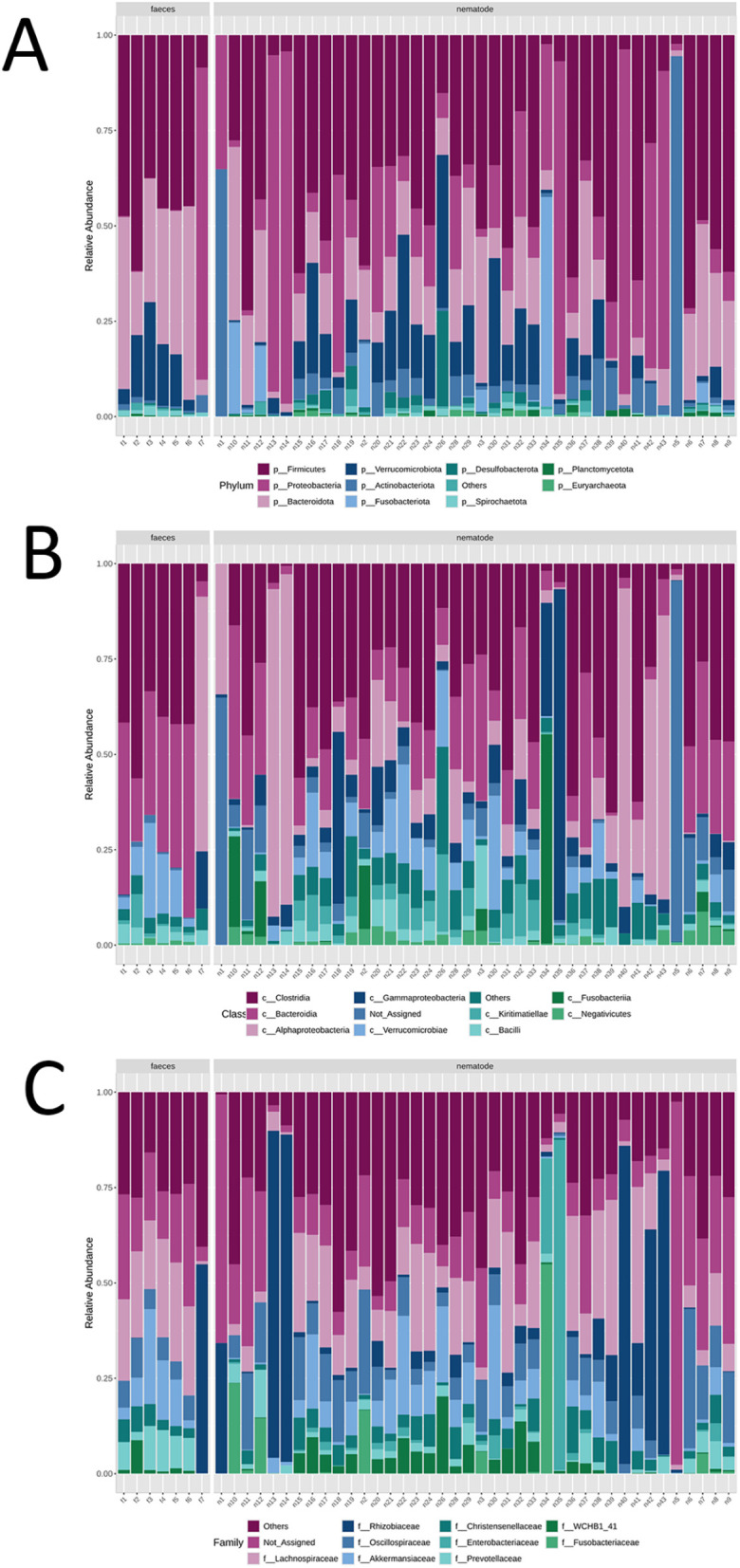
Stacked bar chart illustrating the relative abundance of microbial composition at the phylum (A), class (B), and family (C) levels identified in both faecal and nematode samples at individual level.

The microbial composition at different taxonomic levels (specifically at the phylum, class, and family levels) in faecal and nematode samples are illustrated in Fig 1 and 2.

At the phylum level, Firmicutes (42%), Bacteroidota (32%), Verrucomicrobiota (12%) and Proteobacteria (11%) are predominant in faecal samples (Fig 1), with a notable abundance of Proteobacteria observed in a single sample. Phyla detected in lower proportions include Actinobacteriota, Spirochaetota, Planctomycetota, Desulfobacterota, Euryarchaeota, Chloroflexi, Synergistota, and others. In contrast, nematode samples are characterized by a predominance of Firmicutes (38%) and Proteobacteria (24%), followed by Bacteroidota (15%), Verrucomicrobiota (11%), and, to a lesser extent, Actinobacteriota, Fusobacteriota, Desulfobacterota, Planctomycetota, Spirochaetota, Euryarchaeota, Elusimicrobiota, Halobacterota, Synergistota, and Chloroflexi (Fig 1 and 2). Notably, one of the host samples originated from *Equus asinus* (donkey). Parasite burden and microbiome composition in this individual were comparable to those of the *E. caballus* (horse) samples.

**Fig 3:**
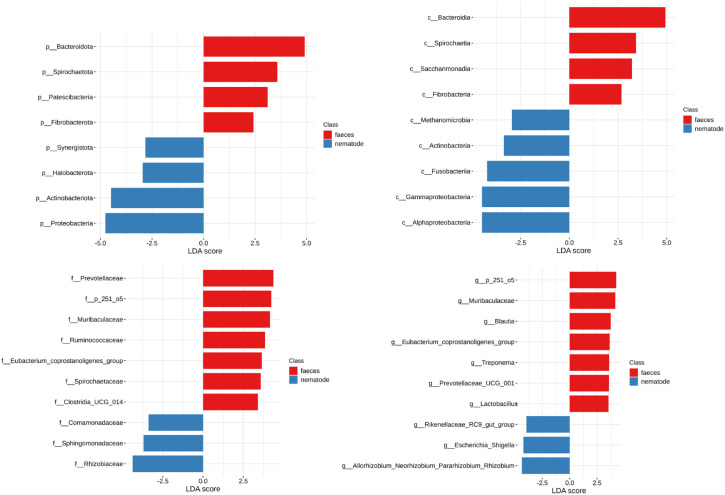
Phyla, classes, families and genera significantly differing between faeces and nematodes, identified through LEfSe analysis and ranked according to their LDA score. Red color: more represented in faeces, blue color more represented in nematodes (less represented in faeces).

At the class level, there is a higher relative abundance of Alphaproteobacteria, Gammaproteobacteria, and Actinobacteria in nematode samples compared to faecal samples (Fig 2). Differences in the relative abundance of various families are also evident. Prevotellaceae, Lactobacillaceae, and Streptococcaceae are more abundant in faecal samples, whereas Rhizobiaceae, Enterobacteriaceae, Rikenellaceae, Moraxellaceae, and Sphingomonadaceae predominate in nematode samples (Fig 3).

**Fig 4:**
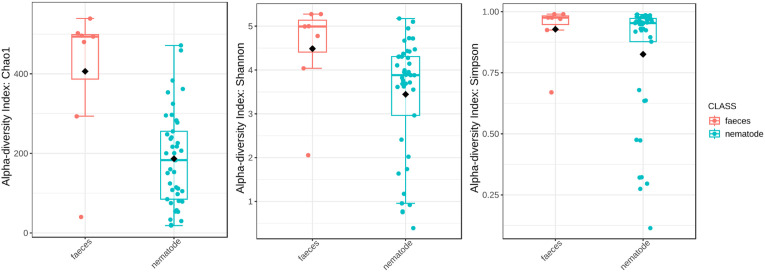
Box-plot chart of alpha diversity according with the Chao1, Shannon and Simpson in faeces and nematodes samples.

**Fig 5:**
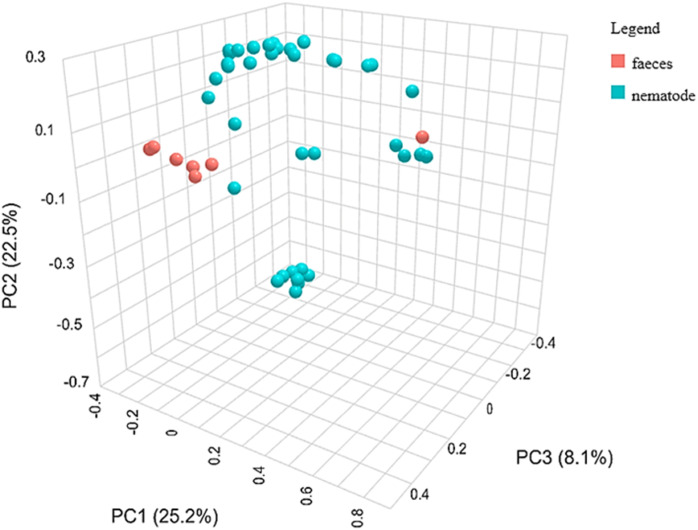
Three-dimensional representation of beta diversity between faeces and nematodes, visualized using PCoA based on Bray-Curtis dissimilarity index and PERMANOVA statistics.

Community profiling through alpha diversity analysis revealed significant differences in microbial richness between the nematode and faecal groups using the Chao1 index (p = 0.004), indicating marked variability in taxonomic richness (Fig 4; Table 2) with faecal samples showing higher richness than nematodes samples. Also, the Shannon (p = 0.008) index showed significant differences, suggesting a lower evenness in nematodes samples despite the Simpson index (p = 0.103) that did not confirm that difference. These findings were further supported by beta diversity analysis using PCoA based on Bray-Curtis dissimilarity, which showed a statistically significant separation between groups (p = 0.007), pinpointing distinct microbial community structures (Fig 5; Table 3).

**Table 2:** Alpha diversity findings between biome (host faeces and nematodes), nematode subfamilies (Cyathostominae and Strongylinae), nematode sex (male vs. female), and intestinal sampling location (caecum vs. ventral colon).

Group comparison	Statistics (Mann-Whitney)	P-value	FDR
**Chao1 index**			
**Faeces vs nematode**	249.0	**0.002**	0.002
**Strongylinae vs Cyathostominae**	227.0	0.533	0.533
**Female vs male**	157.0	0.714	0.714
**Caecum vs ventral colon**	190.0	0.231	0.231
**Shannon index**			
**Faeces vs nematode**	238.0	**0.008**	0.007
**Strongylinae vs Cyathostominae**	132.0	0.066	0.066
**Female vs male**	117.0	0.131	0.131
**Caecum vs ventral colon**	290.0	0.329	0.329
**Simpson index**			
**Faeces vs nematode**	207.0	0.103	0.103
**Strongylinae vs Cyathostominae**	110.0	**0.014**	0.022
**Female vs male**	130.0	0.257	0.257
**Caecum vs ventral colon**	315.0	0.125	0.125

**Table 3:** Beta diversity at feature level between biome (host faeces and nematodes), nematode subfamilies (Cyathostominae and Strongylinae), nematode sex (male vs. female), and intestinal sampling location (caecum vs. ventral colon).

Groups	F-value	R-squared	P-value	FDR
Faeces vs nematode	2.4895	0.050303	0.005	0.005
Strongylinae vs Cyathostominae	6.9865	0.14869	0.001	0.0015
Caecum vs ventral_colon	6.4941	0.1214	0.001	0.001
Female vs male	3.1965	0.074	0.003	0.0045

**Fig 6:**
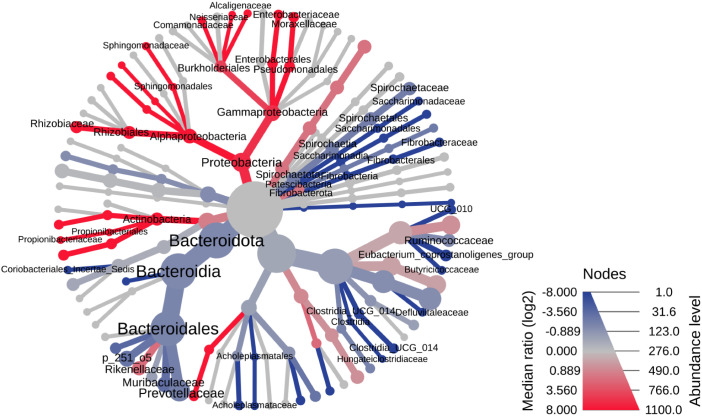
The heat tree analysis highlighting the hierarchical structure of taxonomic classifications to quantitatively (using the median abundance) and statistically (using the non-parametric Wilcoxon Rank Sum test) depict taxonomic differences between microbial communities.

The hierarchical structure analysis of microbiome composition revealed some significant difference in structure and taxa between nematode and faecal samples across the samples as represented in the heat trees highlighting the more or less abundant taxa across the faecal and nematode samples (Fig 6). The heat tree at the phylum level highlights that the phylum Synergistota is significantly more represented in faeces, whereas Proteobacteria and the Alphaproteobacteria (Rhizobiaceae and Sphingomonadaceae), Gammaproteobacteria (Enterobacteriaceae, Moraxellaceae, Neisseriaceae, Moraxellaceae, Comamonadaceae, and Alcaligenaceae) and Actinobacteria (Propionibacteriaceae) classes are significantly more represented in nematodes (Fig 6). Using the LEfSe statistical method similar findings were observed. Nematodes are characterized by a higher abundance of the phylum Proteobacteria and the classes Alphaproteobacteria, Gammaproteobacteria, Actinobacteria, Fusobacteria and Methanomicrobia with higher abundances of Rhizobiaceae, Sphingomonadaceae and Comamonadaceae families (Fig 3).

### Variation in nematode microbiome associated with intestinal localization, strongyle subfamily and host sex

**Fig 7:**
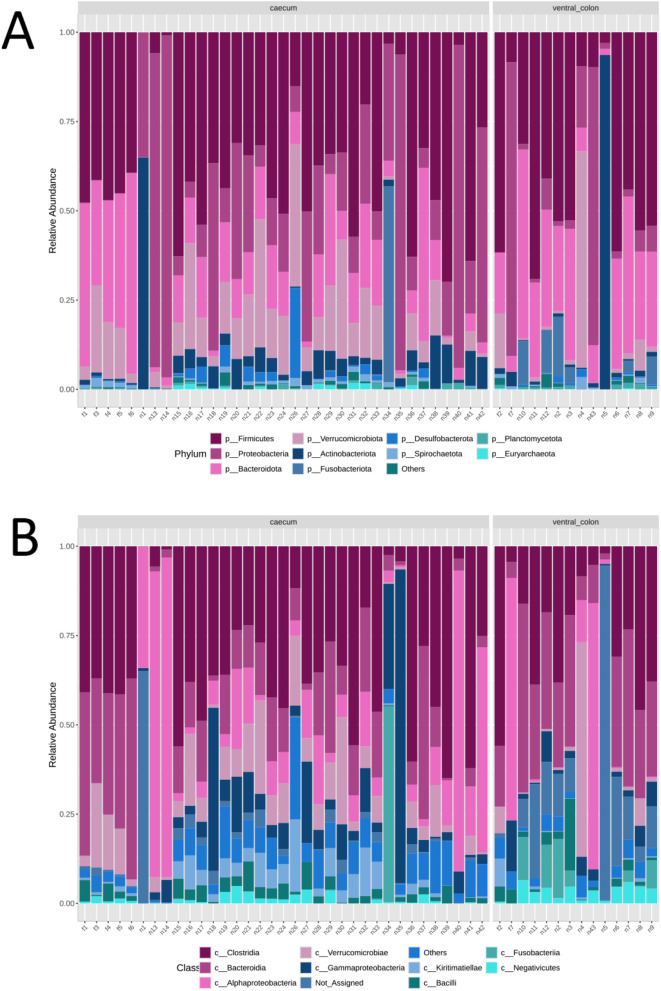
Stacked bar chart illustrating the relative abundance of microbial communities at the phylum (A) and class (B) levels, identified in faeces and in nematodes collected from the caecum and ventral colon at the individual level.

Nematodes from the caecum and ventral colon harbored distinct microbial communities. Caecal nematodes showed higher abundance of Proteobacteria and Verrucomicrobiota, while those from the ventral colon were enriched in Bacteroidota, Fusobacteriota, and Actinobacteriota (Fig 7). At a finer taxonomic resolution, caecal nematodes exhibited higher abundances of Alphaproteobacteria, Kiritimatiellae, Lachnospiraceae, Rhizobiaceae, Akkermansiaceae, and Moraxellaceae, whereas colonic nematodes were characterized by Bacteroidia, Negativicutes, Fusobacteriia, Oscillospiraceae, Puniceicococcaceae, Streptococcaceae, Acidaminococcaceae, and Fusobacteriaceae (S2 Mat).

**Fig 8:**
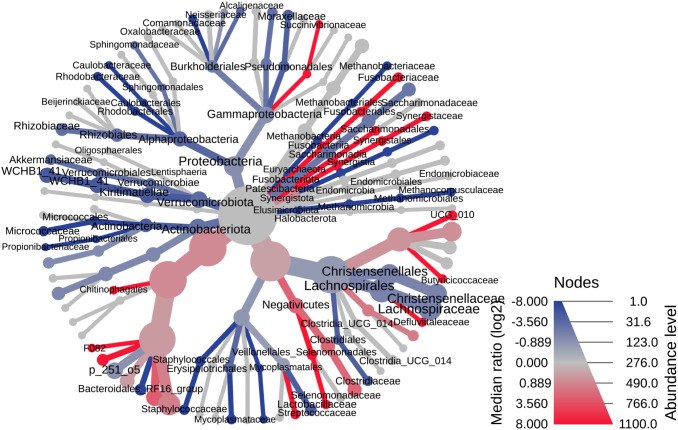
The heat tree analysis highlighting the hierarchical structure of taxonomic classifications to quantitatively (using the median abundance) and statistically (using the non-parametric Wilcoxon Rank Sum test) depict taxonomic differences between microbial communities.

Cyathostominae were enriched in Proteobacteria, Verrucomicrobiota, and Actinobacteriota, while Strongylinae harbored higher abundance of Bacteroidota and Fusobacteria (Fig 8). At the class level, Cyathostominae predominantly contained Alphaproteobacteria, Gammaproteobacteria, Verrucomicrobiae, Kiritimatiellae, and Actinobacteria, whereas Strongylinae were enriched in Bacteroidia, Fusobacteriia, and Negativicutes. Family-level profiles were taxonomically stable within each subfamily: Cyathostominae were characterized by Sphingomonadaceae, Rhodobacteraceae, and Akkermansiaceae, while Strongylinae showed dominance of Prevotellaceae, Fusobacteriaceae, and Streptococcaceae (S Figs). These subfamily-specific compositions suggest a link between nematode taxonomy and microbial community structure.

Both intestinal location and nematode subfamily contributed to microbiome variation. In *Strongylinae*, Proteobacteria predominated in caecal samples while Firmicutes and Bacteroidota were more abundant in colonic samples. However, limited Cyathostominae representation from the colon prevented clear separation of taxonomic versus anatomical effects.

Sex-related differences were observed, with Proteobacteria and Bacteroidota more abundant in females, and Verrucomicrobiota and Actinobacteriota enriched in males. These patterns may be confounded by subfamily, as Strongylinae contained only females while Cyathostominae included both sexes. Males showed higher abundance of Gammaproteobacteria and Actinobacteria at finer taxonomic levels, with greater diversity in dominant families, while Prevotellaceae was female-associated (S Figs).

Alpha diversity metrics showed no significant differences by location, subfamily, or sex, except for Simpson index between Strongylinae and Cyathostominae (p = 0.014). In contrast, beta diversity analysis revealed significant differences in community structure by anatomical site (caecum vs. ventral colon, p = 0.001), subfamily (Cyathostominae vs. Strongylinae, p = 0.001), and sex (p = 0.002) (S Figs).

**Fig 9:**
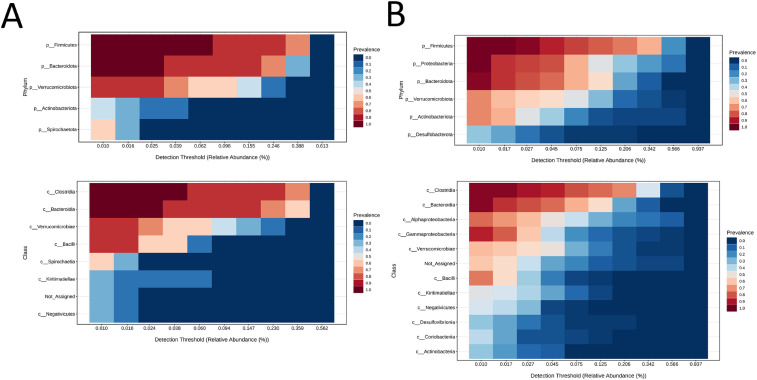
Heatmap showing the core microbiome of faeces (A) and nematodes (B) at the phylum and class levels. Colours ranging from blue to brick red indicate prevalence above specific relative abundance thresholds, as indicated on the x-axis for each taxon listed on the y-axis.

Comparison of equine faecal and nematode microbiomes revealed distinct core communities. At the phylum level, Spirochaetota was exclusive to faeces, while Proteobacteria, Desulfobacterota, and Planctomycetota were unique to nematodes. At the class level, Alphaproteobacteria and Gammaproteobacteria were specific to nematodes. Nematodes exhibited lower alpha diversity and fewer microbial features. Both microbiomes shared Firmicutes (particularly Clostridia and Bacilli), with faeces enriched in Blautia, Ruminococcus, Streptococcus, and Lactobacillus equi, while nematodes were dominated by Proteobacteria including Rhizobium, Sphingomonadaceae, and Moraxellaceae (Fig 9).

**Fig 10:**
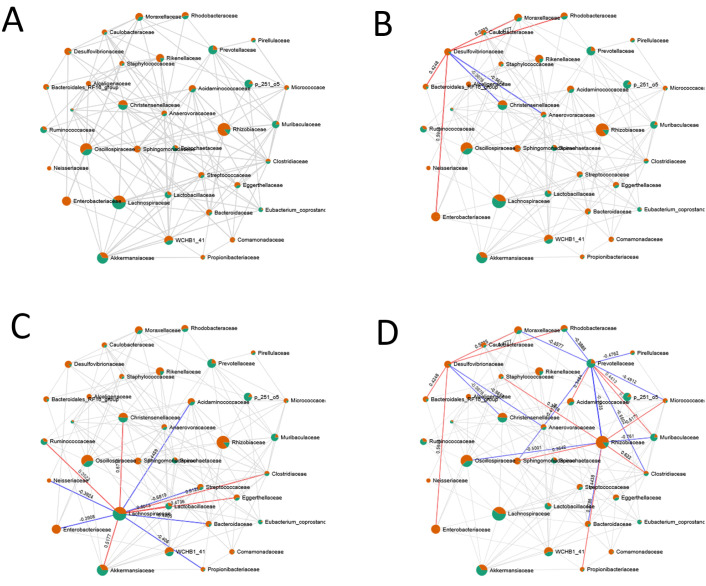
Correlation network analysis. Nodes represent bacterial taxa at the phylum (A) and class (B) levels and are depicted as pie charts, showing relative abundance across faecal (green) and nematode-associated (orange) microbiomes. Edges indicate correlations: red lines represent positive associations; blue lines indicate negative correlations. Only significant correlations were retained for network visualization.

Clustering and correlation network analyses revealed distinct microbial signatures in faeces and nematodes, identifying genera exclusively present in the nematode microbiome, such as Fusobacterium, Afipia, Mesorhizobium, Mycoplasma, and Phoenicibacter. These findings suggest niche-specific associations within the nematode microbiota. The network revealed complex microbial ecological relationships between the intestinal environment and nematodes. Central ecological hubs were identified, representing key taxa either selectively enriched or actively/passively transferred from faeces. Shared taxa suggested microbial exchange or symbiotic associations. Strong positive correlations indicated niche compatibility or mutual support, whereas negative correlations reflected ecological antagonism or niche exclusion (Fig 10; S Figs; Table 4) For example, Clostridia and Bacteroidia dominated the faecal microbiota and showed strong positive associations, consistent with a stable gut environment. In contrast, Gammaproteobacteria, Bacilli, and Actinobacteria were enriched in nematodes, suggesting selective colonization. Negative correlations involving Clostridia, Alphaproteobacteria, Gammaproteobacteria, and Desulfovibrionia supported the hypothesis of ecological exclusion. Planctomycetes were detected in both habitats and positively correlated with Bacilli, indicating potential microbial transfer or co-adaptation. At the family level, Rhizobiaceae, Oscillospiraceae, Sphingomonadaceae, and Enterobacteriaceae were prevalent in nematodes, further supporting selective colonization (S2 Mat; S Figs). Overall, the nematode microbiome appears partially derived from the faecal pool but shaped by selective pressures and ecological filtering (Table 5).

**Table 4:** Correlation network values at the phylum level.

Taxon1	Taxon2	Correlation	P.value	Method
p__Bacteroidota	p__Actinobacteriota	−0.4084	0.004	secom_pearson1
p__Actinobacteriota	p__Bacteroidota	−0.4084	0.004	secom_pearson1
p__Firmicutes	p__Bacteroidota	0.5384	<0.001	secom_pearson1
p__Proteobacteria	p__Bacteroidota	−0.6148	<0.001	secom_pearson1
p__Firmicutes	p__Desulfobacterota	−0.4531	0.005	secom_pearson1
p__Bacteroidota	p__Firmicutes	0.5384	<0.001	secom_pearson1
p__Desulfobacterota	p__Firmicutes	−0.4531	0.005	secom_pearson1
p__Proteobacteria	p__Firmicutes	−0.4224	0.003	secom_pearson1
p__Spirochaetota	p__Firmicutes	−0.3641	0.018	secom_pearson1
p__Verrucomicrobiota	p__Planctomycetota	−0.389	0.028	secom_pearson1
p__Bacteroidota	p__Proteobacteria	−0.6148	<0.001	secom_pearson1
p__Firmicutes	p__Proteobacteria	−0.4224	0.003	secom_pearson1
p__Firmicutes	p__Spirochaetota	−0.3641	0.018	secom_pearson1
p__Verrucomicrobiota	p__Spirochaetota	0.4618	0.003	secom_pearson1
p__Planctomycetota	p__Verrucomicrobiota	−0.389	0.028	secom_pearson1
p__Spirochaetota	p__Verrucomicrobiota	0.4618	0.0027	secom_pearson1

**Table 5:** Examples of ecological interpretations of significant correlations among microbial taxa associated with faeces and nematodes, indicating potential symbiotic, antagonistic, or selective relationships.

Taxon	Predominant in	Key Correlations	Ecological Interpretation
**Clostridia**	Faeces	Negative with *Gammaproteobacteria*	Promotes intestinal eubiosis; antagonistic to potential pathogens
**Bacteroidia**	Faeces	Positive with *Clostridia*	Core members of a stable gut symbiotic community
**Gammaproteobacteria**	Nematodes	Negative with *Clostridia*	Potential opportunistic pathogen; selectively enriched in nematodes
**Bacilli**	Nematodes	Positive with *Planctomycetes*	Possibly adapted to stress-tolerant environments; potential symbiont
**Actinobacteria**	Nematodes	No visible correlations	Likely selected or retained within the nematode microenvironment
**Planctomycetes**	Faeces/Nematodes	Positive with *Bacilli*	Shared commensal between host gut and nematode niche

## Discussion

The key finding of this study is that strongyle nematodes inhabiting the equine gastrointestinal tract possess a distinct, stable microbiome that is taxonomically and functionally different from that of their equine hosts. This divergence indicates selective microbial associations important for parasite survival, adaptation, and pathogenesis.

While equine faecal and nematode microbiomes share certain core phyla (Firmicutes, Bacteroidota, Verrucomicrobiota, and Actinobacteriota) the relative abundances differ significantly. Notably, the nematode microbiome was particularly enriched in Proteobacteria, which was minimally represented in host faeces. These results align with previous findings in ovine and murine nematodes [15–18], supporting the hypothesis of niche-specific microbial selection.

At finer taxonomic resolutions, nematode-associated communities were enriched in Alphaproteobacteria and Gammaproteobacteria, including Rhizobiaceae, Moraxellaceae, and Enterobacteriaceae (e.g., *Escherichia* sp. 4). In contrast, equine faeces were dominated by Lachnospiraceae, Ruminococcaceae, and other Firmicutes-affiliated taxa. The genus *Akkermansia*, particularly *A. muciniphila*, was identified as a key shared component, although it showed higher prevalence in faeces. Its role in mucin degradation and host metabolic regulation [19,20] suggests functional importance in both host and parasite biology.

Alpha diversity analyses demonstrated that the nematode microbiome had lower richness (Chao1 index) but similar evenness compared to the equine gut microbiome. Beta diversity clearly distinguished the two communities, suggesting niche-driven microbial community differentiation. Such patterns may result from anatomical, ecological, and physiological barriers within the parasite, including selective feeding and colonization resistance [16,17].

Nematodes possess a complete digestive system that can host stable microbial communities. While some microbes may be acquired passively from the host environment, current evidence suggests active maintenance and potential vertical transmission of symbiotic bacteria [15,21]. Several genera identified—including *Fusobacterium*, *Escherichia-Shigella*, *Mesorhizobium*, and *Endomicrobium*—are anaerobic bacteria adapted to the hypoxic conditions characteristic of the nematode gut [22]. This pattern reflects broader evolutionary processes seen in host–microbe–parasite systems. As suggested by Drew et al. [23], microbial symbionts can shift over time from harmful to beneficial roles, depending on the host and environmental conditions. In nematodes, this may lead to microbiomes that are shaped by evolution to support parasite survival, reproduction, and immune evasion. These findings show how flexible and adaptive these microbial relationships can be.

The observed stability of the nematode microbiome and the presence of host-independent taxa may suggest, but do not demonstrate, possible vertical transmission or active symbiont maintenance as described in filarial worms harboring *Wolbachia* endosymbionts [21]. Further targeted investigations will be necessary to substantiate this hypothesis.

Interestingly, compartmental differences within the equine GI tract also influenced the nematode microbiome. Strongyles collected from the caecum and ventral colon harbored microbiotas with distinct taxonomic compositions. Proteobacteria and Actinobacteriota were more abundant in caecal nematodes, while Firmicutes and Fusobacteriota dominated the colon-associated microbiomes. Subtle variations in pH, mucus production, and fermentation profiles likely drive microbial filtering in each compartment [24,25].

Sex-based differences were observed in the nematode-associated microbiome. Beta diversity analysis revealed sex-specific microbial compositions. The observed differences may reflect physiological or behavioral differences between male and female parasites, such as gut structure, feeding patterns, or immunological interactions with their microbiota [17].

Differences were also noted between the microbiomes of large and small strongyles (Strongylinae vs. Cyathostominae). Cyathostominae were enriched in Proteobacteria, Verrucomicrobiota, and Actinobacteriota, while Strongylinae showed higher levels of Bacteroidota and Fusobacteriota. These distinctions may reflect their divergent life cycles, feeding strategies, and migratory behaviors [14,26], which shape exposure to environmental microbes and influence microbial acquisition.

The nematode microbiome may contribute to parasite fitness or influence host health through dysbiosis [27,28]. Importantly, proteobacteria dominance in nematodes is often linked to inflammation and dysbiosis in mammals [29,30] and may suggest their potential roles in parasitic pathogenesis.

The identification of a stable, nematode-specific microbiome suggests new strategies for parasite control. Disrupting key symbiotic bacteria using antibiotics [31], probiotics, or engineered bacteriophages [32] could reduce nematode survival. Furthermore, recent evidence suggests that the nematode microbiome itself may influence anthelmintic efficacy, either by modulating host immune responses or through microbial detoxification of drugs. Persistent symbionts such as *Ochrobactrum* and *Stenotrophomonas* [15,17] may interfere with drug action, potentially contributing to reduced susceptibility and the development of resistance. This highlights the need to consider microbial composition when evaluating treatment outcomes. In this context, antibiotics like doxycycline and rifampicin have shown efficacy in targeting endosymbionts in filarial worms, but their broader use in veterinary settings needs caution due to possible effects on the host [33]. To develop safe microbiome-targeted treatments, more research is needed to understand the function of these microbial communities.

In conclusion, this study demonstrates that strongyle nematodes maintain a microbiome distinct from their equine hosts, shaped by anatomical niche, sex, and phylogenetic lineage. These findings support novel, microbiome-informed control strategies, which may prove crucial in combating rising anthelmintic resistance.

## Supporting information

S1 Fig A) Reads per sample and B) Rarefaction curve displaying richness for each sample at 7500 reads per sample. (PDF)

S2 Fig Violin plots reporting phyla statistically different between faeces and nematodes. (PDF)

S3 Fig Violin plots reporting classes statistically different between faeces and nematodes. (PDF)

S4 Fig Correlation network analysis. Nodes represent bacterial taxa at the family level and are depicted as pie charts, showing relative abundance across faecal (green) and nematode-associated (orange) microbiomes. Edges indicate correlations: red lines represent positive associations, blue lines indicate negative correlations. Only significant correlations were retained for network visualization. More connected nodes. Specific correlations are highlighted in: B) Desulfovibrionaceae, Enterobacteriaceae, Christensenellaceae, Anaerovoracaceae; C) Lachnospiraceae, Akkermansiaceae and Enterobacteriaceae and D) Prevotellaceae, Rhizobiaceae, and Oscillospiraceae.
(PDF)

S5 Fig Stacked bar charts showing the relative abundance of microbial composition at the phylum level (top) and class level (bottom). Samples are grouped by anatomical site (caecum and ventral colon, left) and by nematode sex (male and female nematodes, right). Biomes are presented individually and limited to the top 10 taxa.
(PDF)

S6 Fig Heat trees illustrating taxonomic differences between nematodes subfamily (Strongylinae vs Cyatostominae) at the family level. Blue and red colors indicate taxa that are, respectively, less and more abundant in Strongylinae compared to Cyatostominae. Only taxa significantly different at the Wilcoxon Rank Sum test are indicated.
(PDF)

S7 Fig Heat trees illustrating taxonomic differences between nematode sex (female vs male) at the family level. Blue and red colors indicate taxa that are, respectively, less and more abundant in nematodes collected from the caecum compared to those from the ventral colon. Only taxa significantly different at the Wilcoxon Rank Sum test are indicated.
(PDF)

S8 Fig Heat map showing the comparative microbiome composition associated with host and nematodes (biome), nematode subfamilies (Cyathostominae and Strongylinae), nematode sex (male vs. female), and intestinal sampling location (caecum vs. ventral colon). Each row represents a bacterial taxon, while each column corresponds to a sample categorized by subfamily, sex, and intestinal region. Color intensity indicates the relative abundance of each taxon, with warmer colors representing higher abundance levels. Hierarchical clustering reveals similarities and differences in microbial communities across the different biological and anatomical conditions.
(PDF)

S9 Fig Heat map showing the detailed comparative microbiome composition associated with nematode sex (male and female) at class level. Each row represents a bacterial taxon, while each column corresponds to a sample categorized by host faeces and nematode subfamily. Color intensity indicates the relative abundance of each taxon, with warmer colors representing higher abundance levels. Hierarchical clustering reveals similarities and differences in microbial communities across the different groups.
(PDF)

S10 Fig Correlation network analysis at genus level. Nodes represent bacterial taxa at the phylum (A) and class (B) levels and are depicted as pie charts, showing relative abundance across faecal (green) and nematode-associated (orange) microbiomes. Edges indicate correlations: red lines represent positive associations; blue lines indicate negative correlations. Only significant correlations were retained for network visualization.
(PDF)

S2 Mat Correlation Analysis of Microbial Genera–SECOM Pearson1 Output. (XLSX)

S1 Table Distribution of nematode and faecal samples collected per host. (PDF)

S2 Table Summary of Sequencing Reads and Feature Counts per Sample (QIIME2 Output). (XLSX)
