# Supplementary figures and images for: Beyond the host: Unveiling the independent microbiome of equine gastrointestinal nematodes
Source: PLoS One. 2026 Feb 10;21(2):e0339596. doi: 10.1371/journal.pone.0339596 (PMC12890152; doi:10.1371/journal.pone.0339596)

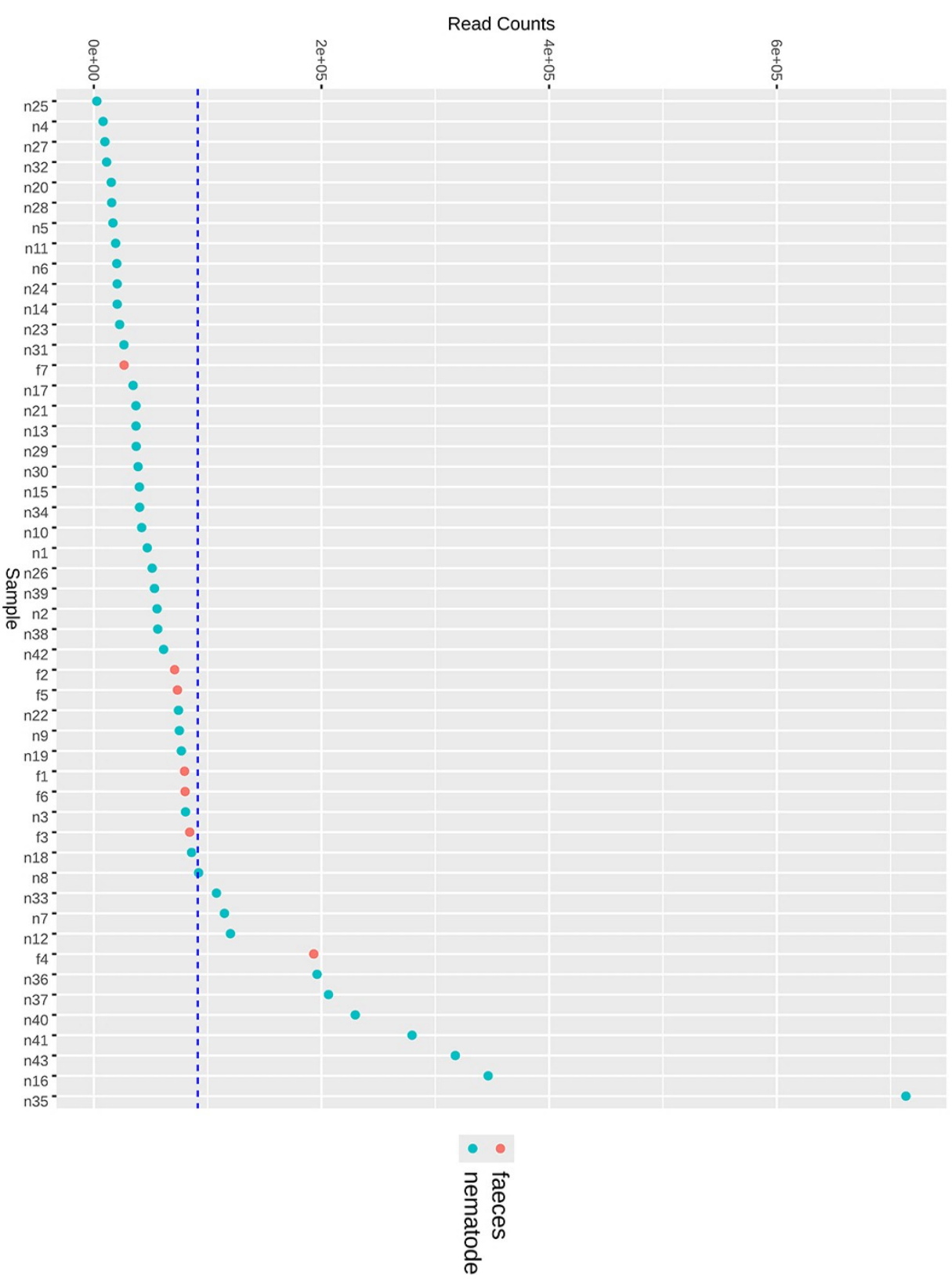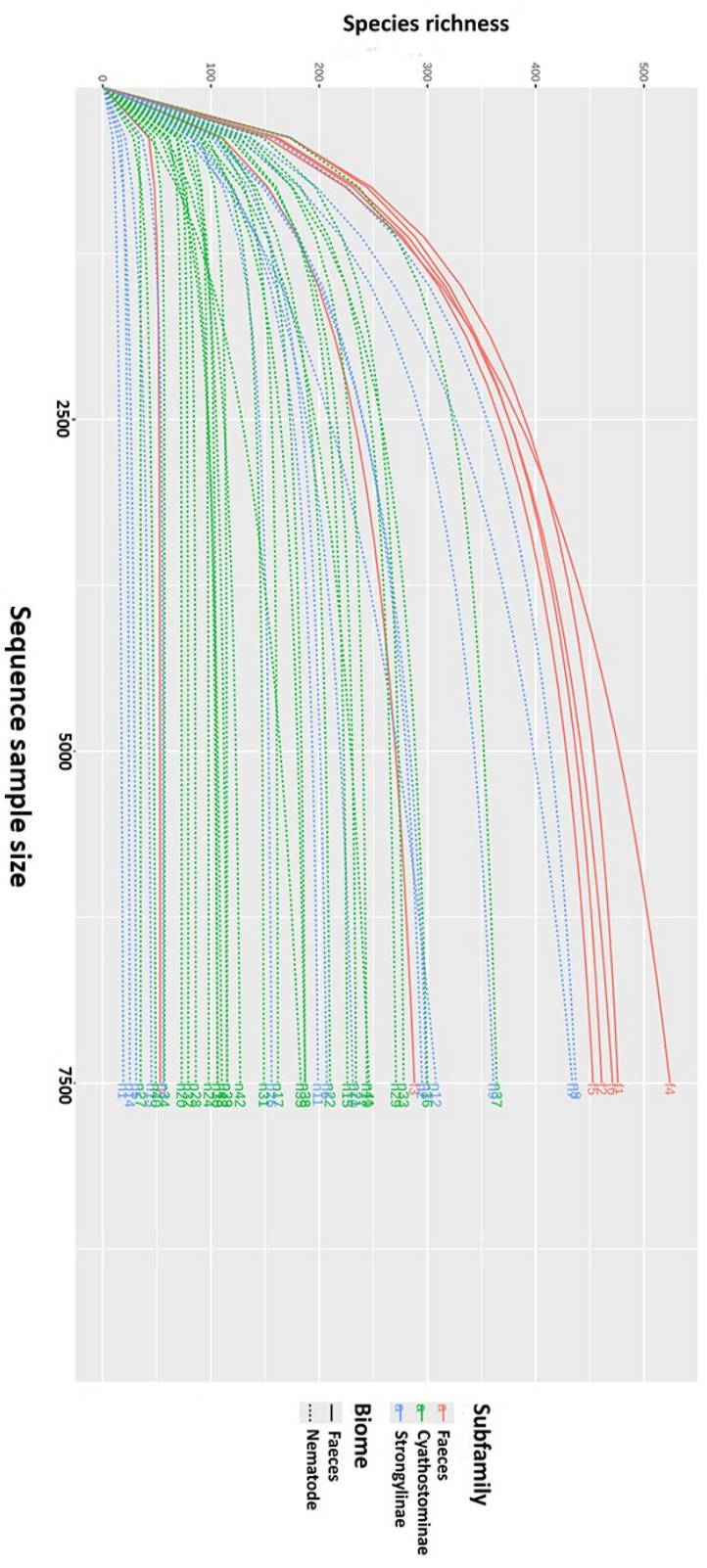

Supplement: S1 Fig — (PDF) [file pone.0339596.s001.pdf]

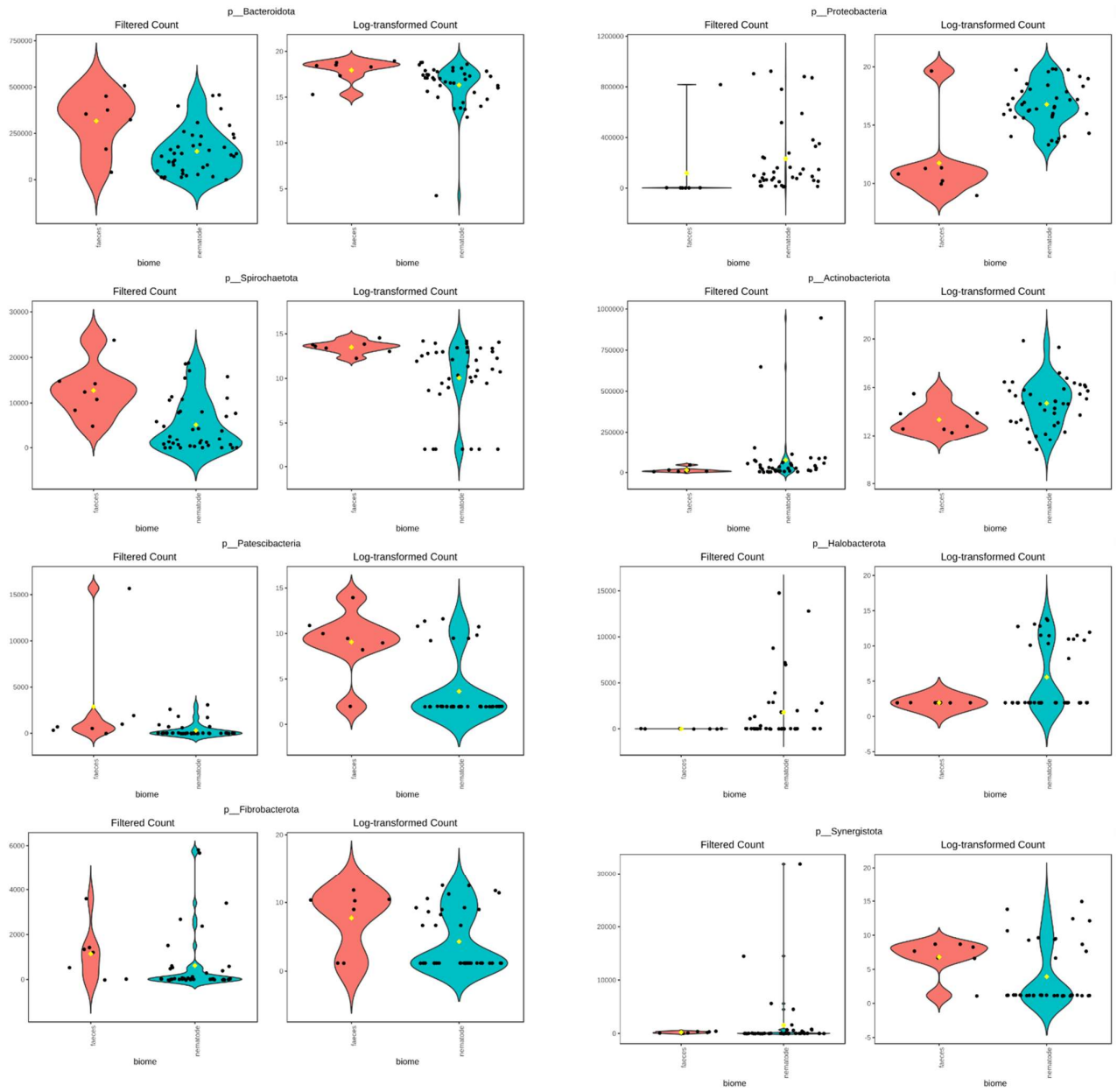

**S1 Fig**

Supplement: S2 Fig — (PDF) [file pone.0339596.s002.pdf]

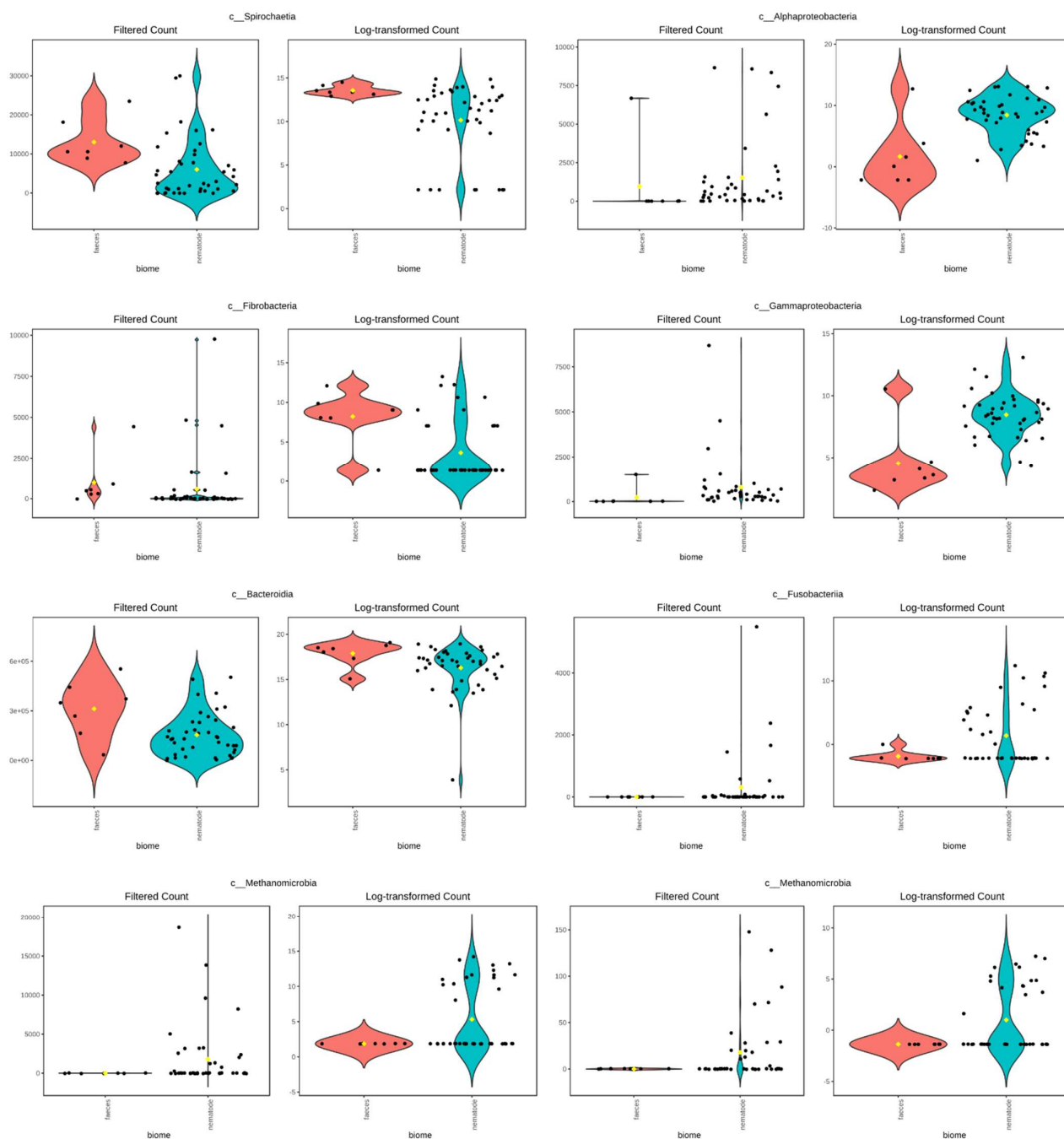

S2 Fig

Supplement: S3 Fig — (PDF) [file pone.0339596.s003.pdf]

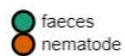

A

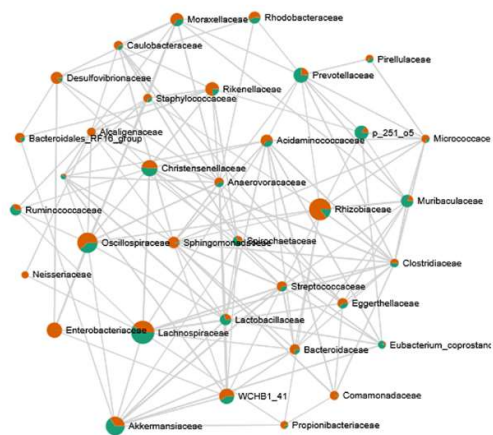

B

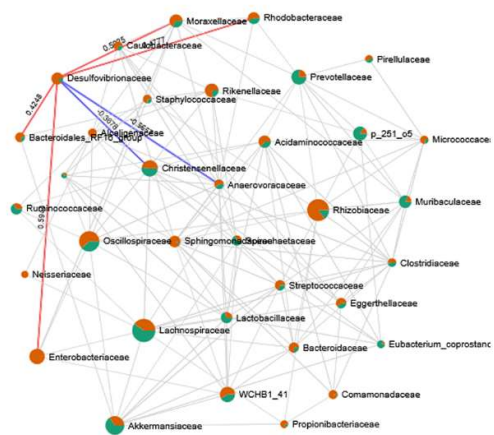

C

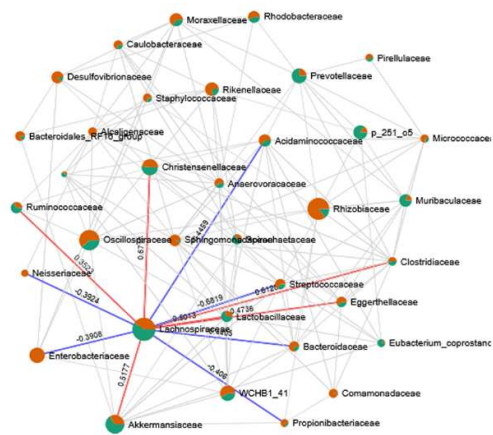

D

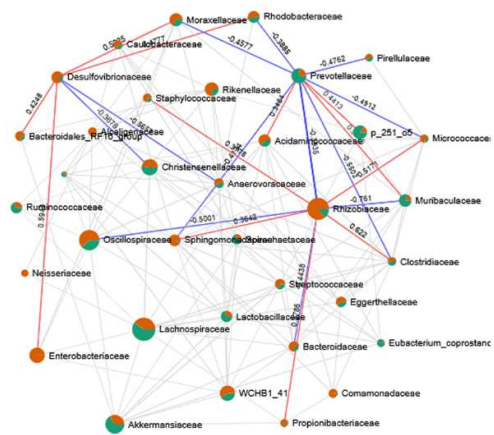

S3 Fig

Supplement: S4 Fig — Nodes represent bacterial taxa at the family level and are depicted as pie charts, showing relative abundance across faecal (green) and nematode-associated (orange) microbiomes. Edges indicate correlations: red lines represent positive associations, blue lines indicate negative correlations. Only significant correlations were retained for network visualization. More connected nodes. Specific correlations are highlighted in: B) Desulfovibrionaceae, Enterobacteriaceae, Christensenellaceae, Anaerovoracaceae; C) Lachnospiraceae, Akkermansiaceae and Enterobacteriaceae and D) Prevotellaceae, Rhizobiaceae, and Oscillospiraceae. (PDF) [file pone.0339596.s004.pdf]

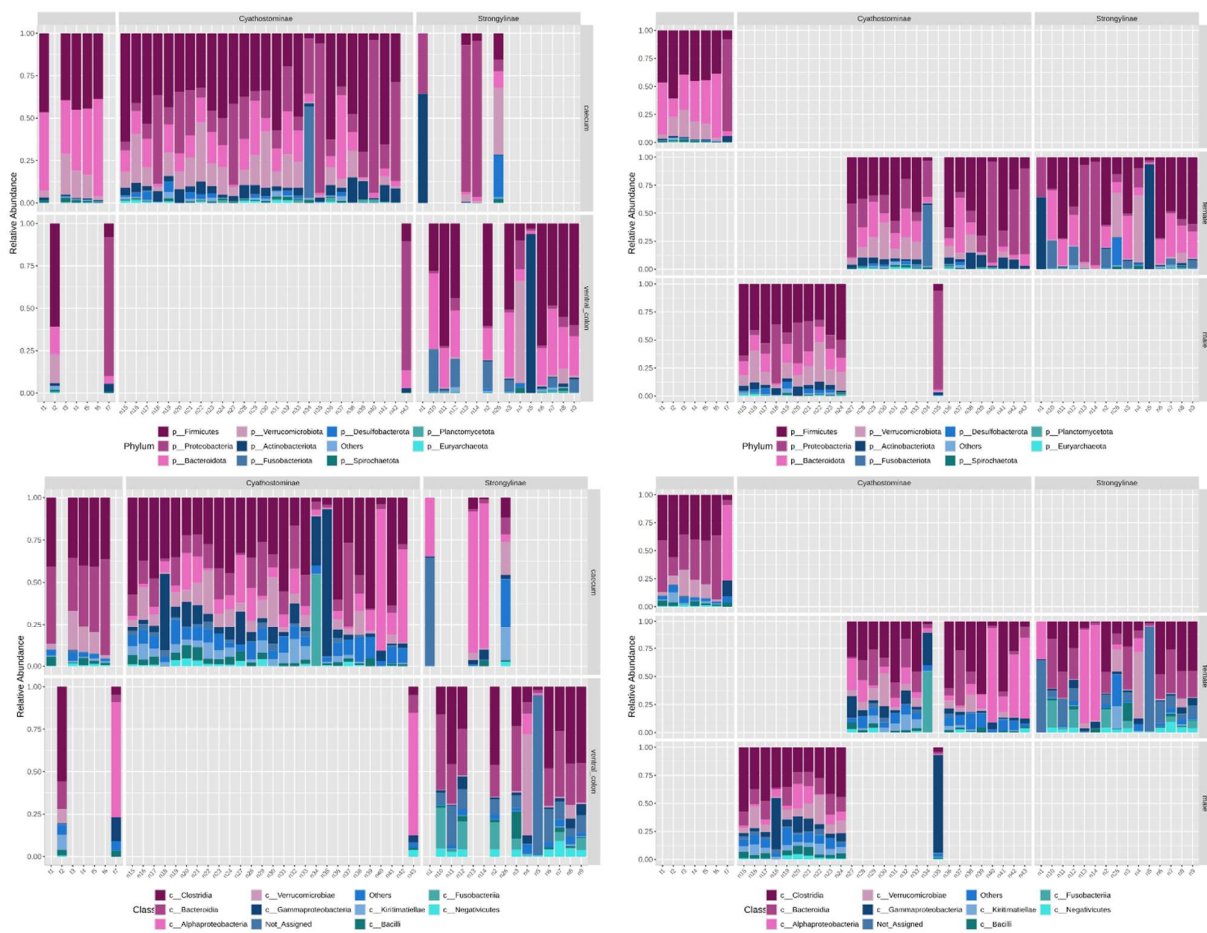

S4 Fig

Supplement: S5 Fig — Samples are grouped by anatomical site (caecum and ventral colon, left) and by nematode sex (male and female nematodes, right). Biomes are presented individually and limited to the top 10 taxa. (PDF) [file pone.0339596.s005.pdf]

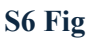

**S6 Fig**

Supplement: S6 Fig — Blue and red colors indicate taxa that are, respectively, less and more abundant in Strongylinae compared to Cyatostominae. Only taxa significantly different at the Wilcoxon Rank Sum test are indicated. (PDF) [file pone.0339596.s006.pdf]

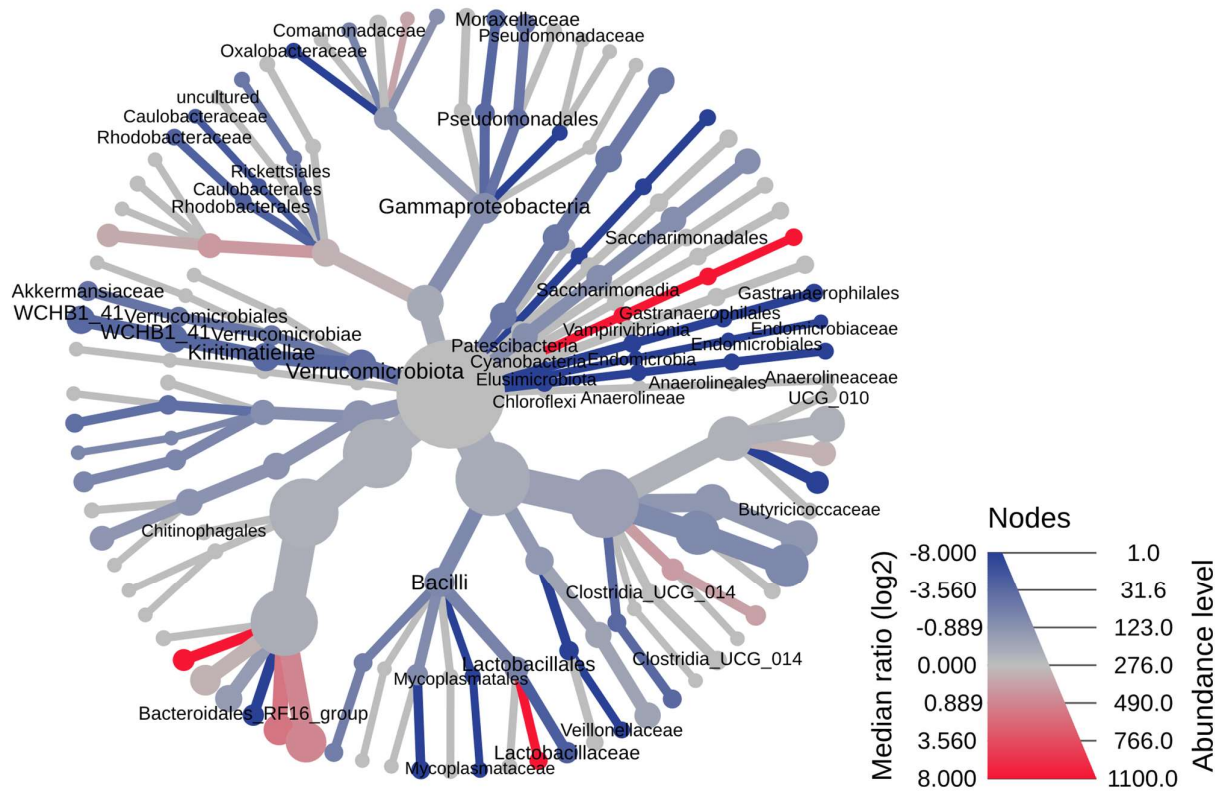

S7 Fig

Supplement: S7 Fig — Blue and red colors indicate taxa that are, respectively, less and more abundant in nematodes collected from the caecum compared to those from the ventral colon. Only taxa significantly different at the Wilcoxon Rank Sum test are indicated. (PDF) [file pone.0339596.s007.pdf]

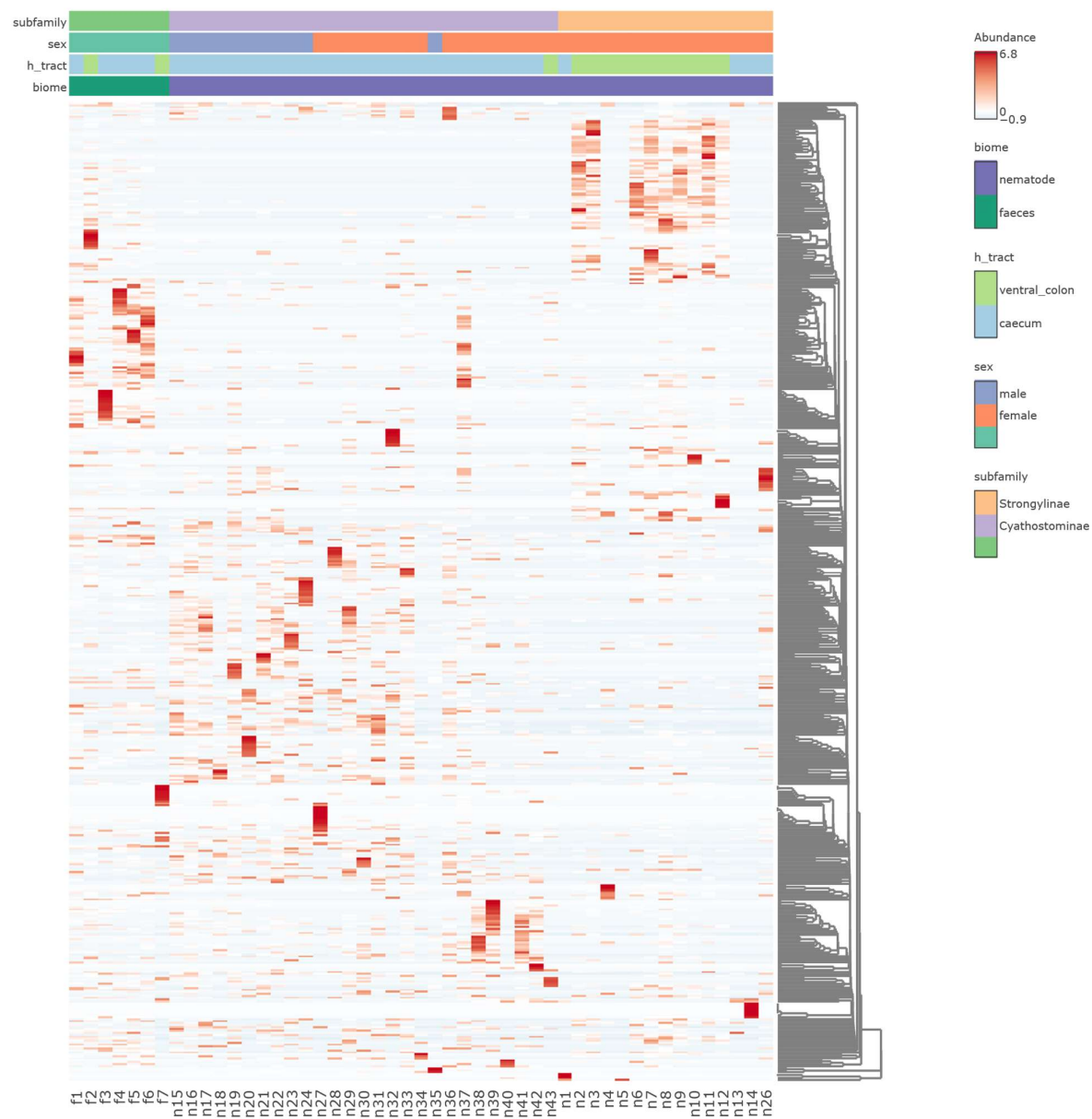

**S8 Fig**

Supplement: S8 Fig — Each row represents a bacterial taxon, while each column corresponds to a sample categorized by subfamily, sex, and intestinal region. Color intensity indicates the relative abundance of each taxon, with warmer colors representing higher abundance levels. Hierarchical clustering reveals similarities and differences in microbial communities across the different biological and anatomical conditions. (PDF) [file pone.0339596.s008.pdf]

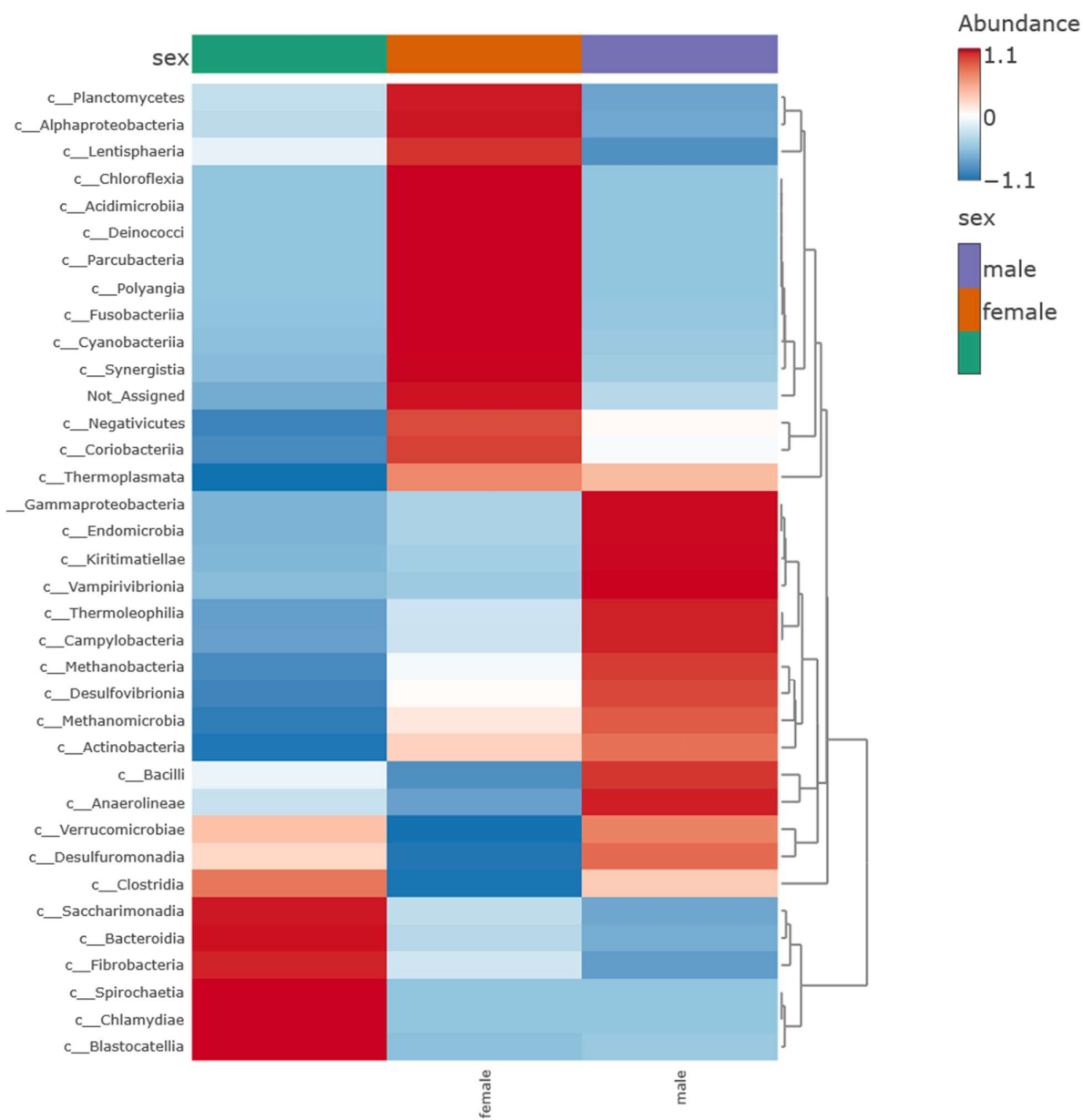

**S10 Fig**

Supplement: S9 Fig — Each row represents a bacterial taxon, while each column corresponds to a sample categorized by host faeces and nematode subfamily. Color intensity indicates the relative abundance of each taxon, with warmer colors representing higher abundance levels. Hierarchical clustering reveals similarities and differences in microbial communities across the different groups. (PDF) [file pone.0339596.s009.pdf]
