# Supplementary material for: Beyond the host: Unveiling the independent microbiome of equine gastrointestinal nematodes
Source: PLoS One. 2026 Feb 10;21(2):e0339596. doi: 10.1371/journal.pone.0339596 (PMC12890152; doi:10.1371/journal.pone.0339596)
Supplement: S1 Table — (PDF) [file pone.0339596.s012.pdf]

S2 Table.

Host ID	Species	Nematode samples(n)	Faecal samples(n)
1	*Equus caballus*	1	1
2	*Equus caballus*	11	1
3	*Equus caballus*	1	1
4	*Equus caballus*	1	1
5	*Equus asinus*	20	1
6	*Equus caballus*	9	2
