# Supplementary material for: Beyond the host: Unveiling the independent microbiome of equine gastrointestinal nematodes
Source: PLoS One. 2026 Feb 10;21(2):e0339596. doi: 10.1371/journal.pone.0339596 (PMC12890152; doi:10.1371/journal.pone.0339596)
Supplement: S10 Fig — Nodes represent bacterial taxa at the phylum (A) and class (B) levels and are depicted as pie charts, showing relative abundance across faecal (green) and nematode-associated (orange) microbiomes. Edges indicate correlations: red lines represent positive associations; blue lines indicate negative correlations. Only significant correlations were retained for network visualization. (PDF) [file pone.0339596.s010.pdf]

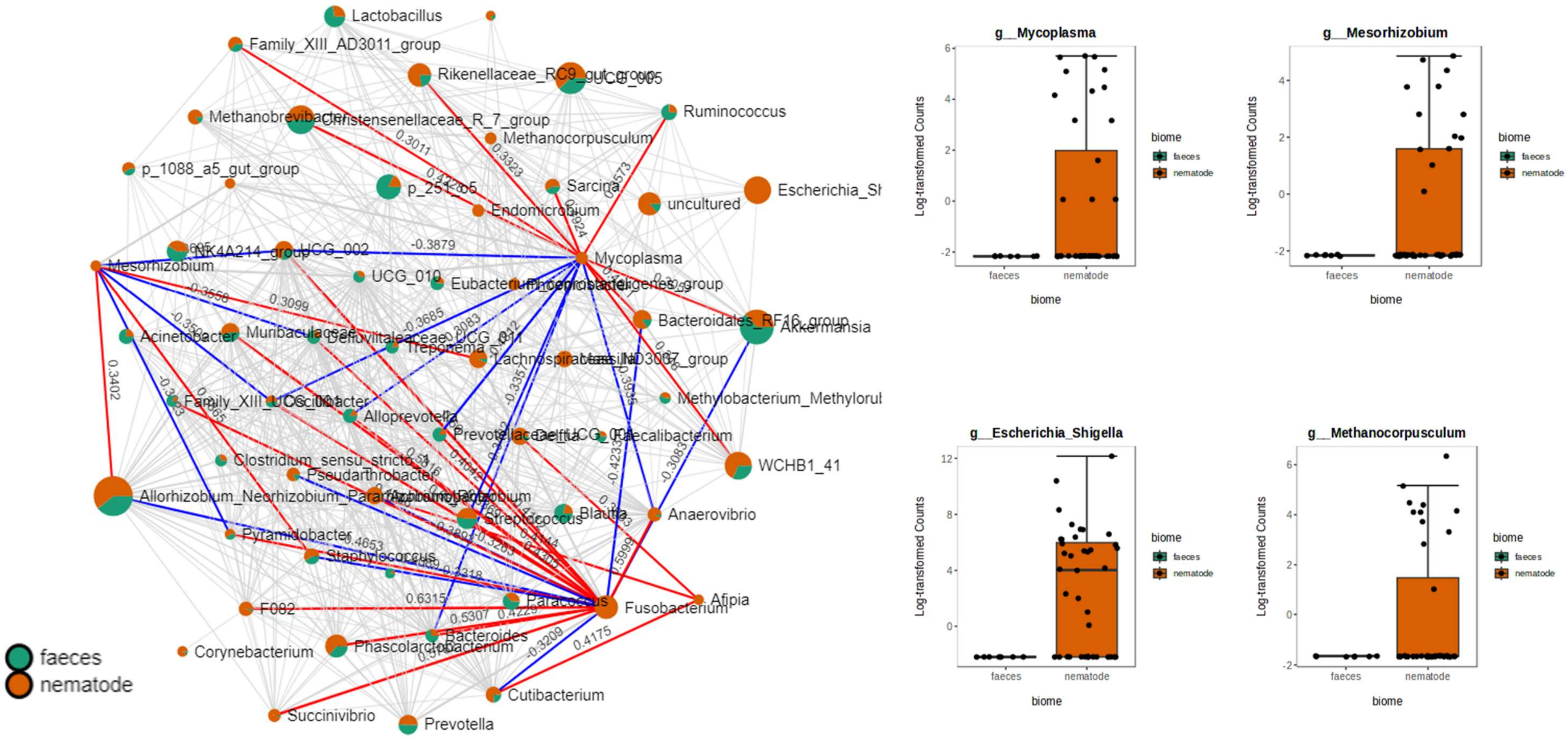
Lactobacillus
Family_XIII_AD3011_group
Ruminococcus
Methanocorpusculum
p_1088_a5_gut_group
Sarcina
Escherichia_Sh
uncultured
Endomicrobium
Mycoplasma
Mesorhizobium
UCG_010
Acinetobacter
Methylobacterium_Methylorul
Alloprevotella
WCHB1_41
Clostridium_sensu_stricto_1
Pyramidobacter
Anaerovibrio
Staphylococcus
Afipia
F082
Fusobacterium
Corynebacterium
Cutibacterium
Succinivibrio
Prevotella
faeces
nematode
g__Mycoplasma
g__Mesorhizobium
g__Escherichia_Shigella
g__Methanocorpusculum
Log-transformed Counts
biome
faeces
nematode

S12 Fig
